# Renoprotective effect of SGLT-2 inhibitors among type 2 diabetes patients with different baseline kidney function: a multi-center study

**DOI:** 10.1186/s12933-021-01396-2

**Published:** 2021-10-07

**Authors:** Fang-Ju Lin, Chi-Chuan Wang, Chien-Ning Hsu, Chen-Yi Yang, Chih-Yuan Wang, Huang-Tz Ou

**Affiliations:** 1 School of Pharmacy, College of Medicine, National Taiwan University, Taipei, Taiwan; 2 Graduate Institute of Clinical Pharmacy, College of Medicine, National Taiwan University, Taipei, Taiwan; 3 Department of Pharmacy, National Taiwan University Hospital, Taipei, Taiwan; 4 Department of Pharmacy, Kaohsiung Chang Gung Memorial Hospital, Kaohsiung, Taiwan; 5 College of Pharmacy, Kaohsiung Medical University, Kaohsiung, Taiwan; 6 Institute of Clinical Pharmacy and Pharmaceutical Sciences, College of Medicine, National Cheng Kung University, Tainan, Taiwan; 7 College of Medicine, National Taiwan University, Taipei, Taiwan; 8 Department of Internal Medicine, National Taiwan University Hospital, Taipei, Taiwan; 9 Department of Pharmacy, College of Medicine, National Cheng Kung University, Tainan, Taiwan

**Keywords:** Sodium glucose cotransporter-2 inhibitors, Estimated glomerular filtration rate, Kidney function, Type 2 diabetes

## Abstract

**Background:**

To assess the effect of sodium glucose cotransporter-2 inhibitors (SGLT-2is) for type 2 diabetes on kidney outcomes stratified by patient baseline estimated glomerular filtration rate (eGFR) levels (i.e., eGFR ≤ 60, 60 < eGFR ≤ 90, and eGFR > 90 mL/min/1.73 m^2^).

**Methods:**

Patients from three large healthcare delivery systems in Taiwan who had initiated SGLT-2is or other glucose-lowering drugs (oGLDs) between May 2016 and December 2017 were included. Main outcomes were the times to 30%, 40%, and 50% eGFR reduction after treatment initiation. One-to-one propensity score matching in the overall study cohort and in each eGFR subgroup between SGLT-2i and oGLD users was applied to ensure between-group comparability in baseline characteristics.

**Results:**

There were 13,666 matched pairs of SGLT-2is and oGLD users in the overall cohort. While a sustained eGFR decline was revealed in oGLD-treated patients (mean values [standard errors] from 85.61 [0.43] to 82.49 [0.44] mL/min/1.73 m^2^ during the 12 months after treatment initiation), the mean eGFR values of SGLT-2i users decreased in the first 3 months (85.68 [0.37] to 79.71 [0.41] mL/min/1.73 m^2^) but then improved and sustained until the end of follow-up. There were 2300, 5705, and 5509 matched SGLT-2i and oGLD users in the eGFR ≤ 60, 60 < eGFR ≤ 90, and eGFR > 90 subgroups, respectively. Using SGLT-2is versus oGLDs was significantly associated with slower eGFR declines; hazard ratios (HRs) were 0.51 (95% CI 0.37–0.69), 0.51 (0.37–0.70), and 0.47 (0.31–0.71) for 40% eGFR reduction in the eGFR ≤ 60, 60 < eGFR ≤ 90, and eGFR > 90 subgroups, respectively. The renoprotective effect of SGLT-2is versus oGLDs was confirmed in the outcomes of 30% and 50% eGFR reduction across the three eGFR subgroups.

**Conclusions:**

This study supports the renoprotective benefit of real-world SGLT-2i use irrespective of patient baseline kidney function.

**Supplementary Information:**

The online version contains supplementary material available at 10.1186/s12933-021-01396-2.

## Background

Renoprotective benefits associated with sodium glucose cotransporter-2 inhibitor (SGLT-2i) use have been reported in large-scale cardiovascular outcome trials (CVOTs) based on the secondary analysis of the composite microvascular outcome [1–4]. The kidney benefits of SGLT-2i therapy include the alleviation of albuminuria and the mitigation of the deterioration of kidney function. Recently, the CREDENCE trial [5], the first dedicated trial to report a definitive kidney effect of SGLT-2i use, provided the first robust evidence for the kidney benefits of canagliflozin. In the United States, canagliflozin became the first approved SGLT-2i with an indication for the prevention of kidney failure for type 2 diabetes. As extending the body of evidence on the marked renal benefit of SGLT2is for type 2 diabetes, the DAPA-CKD trial [6] further evaluated dapagliflozin use in a chronic kidney disease (CKD) population that comprised patients without type 2 diabetes and with broader renal inclusion criteria. The results of DAPA-CKD support that, in addition to conferring renal benefit in patients with type 2 diabetes and albuminuric CKD, SGLT2is also play a role in the prevention of CKD progression in the absence of diabetes [6]. However, these clinical trials generally enroll highly selected patient populations [e.g., type 2 diabetes with established cardiovascular diseases (CVDs) or existing CVD risk factors in CVOTs [1–3], type 2 diabetes with CKD in the CREDENCE trial [5]]; therefore, the results might not directly reflect treatment effectiveness in real-world settings. In addition, based on recent recommendations [7, 8], it remains unclear whether SGLT-2i use can yield clearly favorable kidney protection due to the different patient characteristics across the trials [1–3, 5, 6].

Kidney outcomes for real-world SGLT-2i use have been reported [9–17]. Studies that include a large, broad type 2 diabetes patient population treated in routine practice are essential for determining whether the renal benefits of SGLT-2is found in trial settings can be translated to clinical practice and quantifying the magnitude of SGLT-2i effectiveness. However, few real-world studies have reported laboratory measures associated with patient kidney function (e.g., estimated glomerular filtration rate [eGFR]) [11–13, 15]. One such study is Comparative Effectiveness of Cardiovascular Outcomes in New Users of SGLT2 Inhibitors 3 (CVD-REAL 3) [13], which included more than 71,000 type 2 diabetes patients across five countries. It provided supporting evidence for the real-world kidney outcomes of SGLT-2i use and suggested that the kidney benefits reported in clinical trials can be extended to clinical practice settings [13]. However, CVD-REAL 3 included only about 8000 patients from Western Pacific countries (i.e., Japan and Taiwan), and approximately 63,000 patients from Israel, the United Kingdom, and Italy [13]. Therefore, there was large uncertainty in the study estimates for Western Pacific countries; nevertheless, the direction of kidney benefits across the countries seemed consistent [13]. In addition, although the mean annual eGFR changes stratified by patient baseline eGFR levels were reported in CVD-REAL 3 [13], the change in eGFR over time was not clearly shown. Further analysis is warranted to assess the effect of SGLT-2i on eGFR in patients with different baseline kidney function.

The present study includes a large number of type 2 diabetes patients from three large healthcare delivery systems in Taiwan to extend existing real-world evidence to a larger Asian population and provide further evidence from patient subgroups with different baseline eGFR levels (i.e., eGFR ≤ 60, 60 < eGFR ≤ 90, and eGFR > 90 mL/min/1.73 m^2^).

## Methods

### Data source

This study was conducted using the electronic health records (EHRs) from three healthcare delivery systems in Taiwan, namely the Integrated Medical Database of National Taiwan University Hospital (NTUH-iMD), the Chang Gung Research Database (CGRD), and the EHR database of National Cheng Kung University Hospital (NCKUH). Briefly, the NTUH-iMD contains individual patient-level medical records from National Taiwan University Hospital (NTUH), a 2554-bed medical center in northern Taiwan. The CGRD contains individual EHRs from the Chang Gung Memorial Hospitals (CGMH) network, with a total of 9584 beds from two medical centers, two regional hospitals, and three district hospitals across Taiwan. NCKUH is a medical center with 1331 beds located in southern Taiwan. These healthcare delivery systems account for approximately 17% of healthcare services reimbursed by Taiwan’s National Health Insurance (NHI) program. Data from May 2016 to December 2017 were used for analysis.

To facilitate multi-database analyses, the three EHR databases were first transformed into a study-specific common data model (CDM) at an individual patient level [18]. Specifically, we developed a common protocol and identified key data elements agreed by the three study sites; next, data intended to be used in this study were transformed into the CDM at each local site (i.e., NTUH, CGMH, and NCKUH). The study-specific CDM followed the data structure of the Sentinel CDM version 6.0.2 in the United States [19]. The following tables were constructed: Demographic, Encounter, Diagnosis, Laboratory Result, and Vital Signs. Considering that dispensing information may be incomplete in our EHR databases, we constructed a Prescribing table containing more comprehensive patient medication records to replace the Dispensing table in the Sentinel CDM. We transformed only relevant EHR data of type 2 diabetes patients into the CDM; for example, for laboratory results, only total cholesterol, creatinine, and hemoglobin A1c (HbA1c) were transformed into the CDM Laboratory Result Table. Detailed elements of the CDM are presented in Additional file 1: Figure S1. After completing CDM construction, all study analyses were performed through a distributed data network. Specifically, detailed analyses were conducted at the local study sites using common, distributed analytic programming, and only aggregated results were sent back to the study coordination center, located at National Cheng Kung University, for further pooled analyses.

### Cohort identification

Using the three EHR databases, we first identified new users of SGLT-2is and other glucose-lowering drugs (oGLDs) at the outpatient department. Only two SGLT-2is, dapagliflozin and empagliflozin, were available in the three study healthcare delivery systems during the study period. The oGLDs included metformin, sulfonylureas, metiglinides, dipeptidyl peptidase-4 (DPP-4) inhibitors, thiazolidinediones, acarbose, glucagon-like peptide-1 (GLP-1) receptor agonists, and insulins. To qualify as new users of a certain medication class, patients had to have not received that class of drug for at least one year prior to the date of initiation (i.e., index date). Other inclusion criteria were an age of 20 years or older at the index date, having at least one year of baseline history in the EHR database prior to the index date, and having at least two pre-index measurements of eGFR. The last pre-index eGFR measurement was required to be within 180 days prior to the index date, and the first and last pre-index measurements were required to be at least 180 days apart. We collected patient baseline eGFR data up to four years before the index date. The pre-index eGFR measurements were modeled using linear regression analyses to estimate the pre-index eGFR change (i.e., slope) and predict the baseline eGFR value at the index date (i.e., intercept) at the individual patient level. Patients were excluded if they had type 1 diabetes or gestational diabetes in the year before the index date. Patients could be included more than once in the analysis as long as their new episodes of drug use met all the inclusion and exclusion criteria. The design for cohort identification and the study variables were generally aligned with the methods employed in CVD-REAL 3 [13].

### Outcomes, follow-up, and study covariates

The outcomes of interest were: (1) change in eGFR after SGLT-2i or oGLD initiation and (2) time to 30%, 40%, and 50% eGFR reduction after SGLT-2i or oGLD initiation.

Using the intention-to-treat (ITT) analytic approach, patients were followed up from the index date until the occurrence of study outcomes, last encounter date in the EHR database, last date of the study period (December 31, 2017), or death, whichever came first. Using the alternative on-treatment (OT) approach (sensitivity analysis), patients were followed up from the index date until the occurrence of study outcomes, discontinuation of index medication, switch to or addition of other type of oGLD, last encounter date in the EHR database, last date of the study period, or death, whichever came first.

The following study covariates were measured at the baseline and treated as potential confounders to be adjusted in the analyses: age, gender, body mass index, smoking, HbA1c, eGFR, eGFR change (slope), frailty status, prior use of oGLDs and other medications (e.g., antihypertensive drugs, aldosterone antagonists, antiplatelets, and statins), which were measured in the year before the index date, and history of microvascular diseases and CVDs (i.e., myocardial infarction, unstable angina, heart failure, atrial fibrillation, stroke, and peripheral artery disease), which were based on all available diagnosis records in the EHR databases before the index date. In the absence of a gold standard, the baseline status of frailty in this study was operationally defined as having one or more hospitalizations for at least 3 consecutive days in the year prior to the index date. For baseline laboratory values (e.g., HbA1c), only the last measurement in the year prior to the index date was included.

### Statistical analyses

Propensity score (PS) matching was applied to balance differences in baseline demographic and clinical characteristics between the SGLT-2i and oGLD groups. The PS (the probability of initiating SGLT-2is versus oGLDs) was estimated by logistic regressions with the aforementioned covariates. SGLT-2i and oGLD users were matched at a 1:1 ratio within each healthcare delivery system. The matching was performed using the nearest neighbor method with a caliper width of 0.25 multiplied by the standard deviation of the PS distribution [20]. Baseline characteristics were compared between the treatment groups before and after matching. The between-group comparability in baseline characteristics was assessed using the standardized mean difference.

Importantly, to assess the effect of SGLT-2is for patients with different baseline kidney function, we performed a stratified analysis using eGFR levels in addition to the analysis of the overall cohort. Patients were first classified into three subgroups according to their baseline eGFR values: eGFR ≤ 60, 60 < eGFR ≤ 90, and eGFR > 90 mL/min/1.73 m^2^ [13]. eGFR at the baseline was calculated based on a mean serum creatinine (SCr) level retrieved within 3 months prior to the index date using the Modification of Diet in Renal Disease equation [21]: 175 × SCr (mg/dL)^−1.154^ × Age (years)^−0.203^ × 0.742 (if female). To ensure that patient characteristics were well-balanced between treatment groups within each eGFR subgroup, the PS was re-estimated (using logistic regression analyses) for each eGFR subgroup, and SGLT-2i and oGLD users were re-matched within each eGFR strata. The outcomes of interest were then evaluated for each eGFR subgroup.

Among the matched SGLT-2i or oGLD users, we first calculated incidence rates of study outcomes, which are expressed as per 100 person-years with 95% CIs. The changes in eGFR values after the index date were calculated. We estimated the hazard ratios (HRs) of 30%, 40%, and 50% eGFR reduction using Cox proportional hazards models to compare the risk of kidney function deterioration between SGLT-2i or oGLD users. All analyses abovementioned were conducted using SAS software (version 9.4; SAS Institute, Cary, NC).

### Meta-analysis procedures

Once the results of study outcomes were available from individual healthcare delivery systems, a meta-analysis was conducted to pool the results across the systems to summarize the estimates. Even though data from different healthcare delivery systems were transformed into a CDM, the generic inverse-variance method with random effects in the meta-analysis was applied because heterogeneity in patient characteristics across individual health systems might still exist [22]. The random-effects approach assumes that a normal distribution of effects exists and is weighted with both the within- and between-studies variances, resulting in a more conservative estimate with a wider CI. RevMan5 (Nordic Cochrane Centre, Copenhagen, Denmark) software was used for the meta-analysis.

## Results

**Fig. 1:**
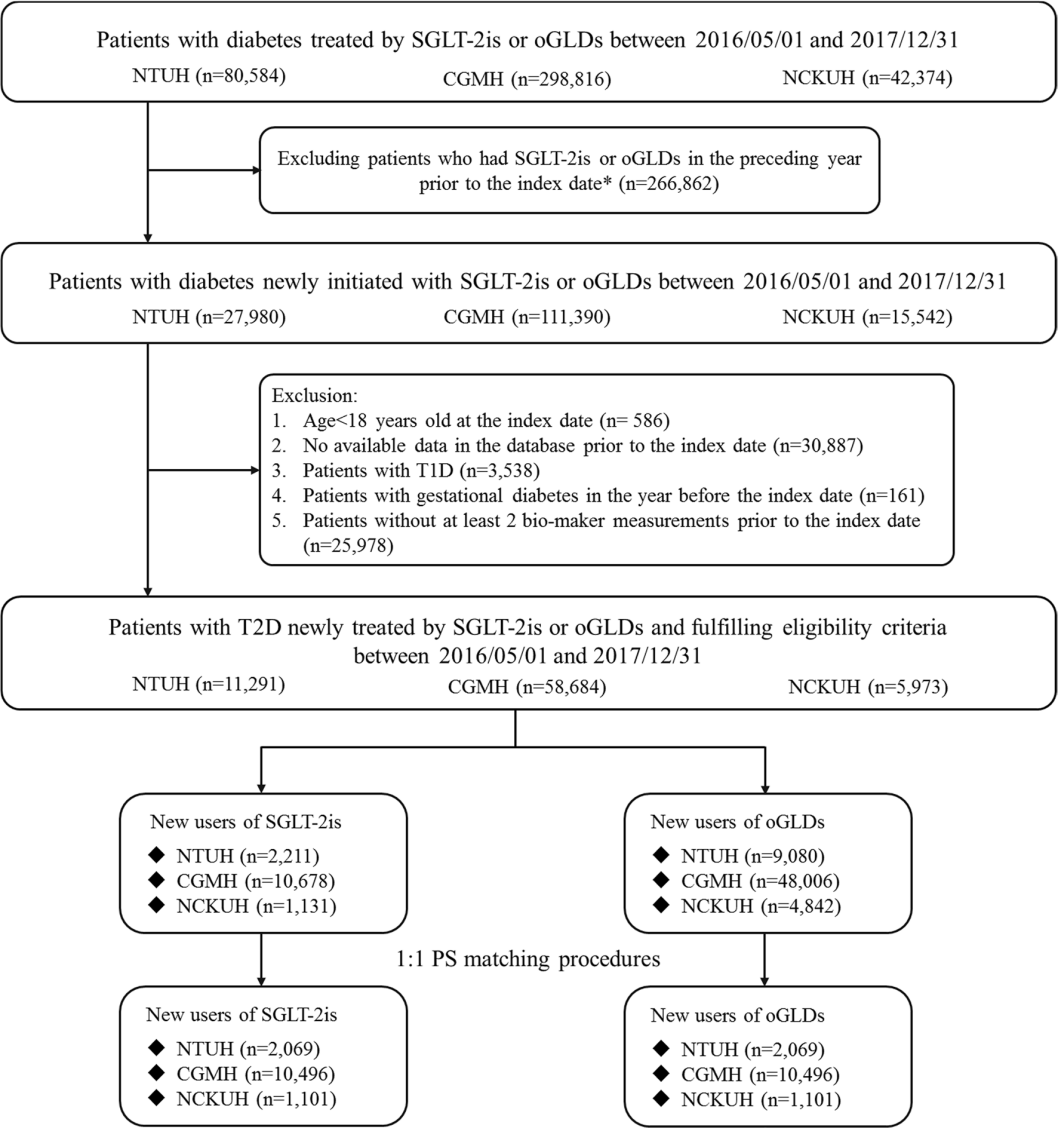
Flow chart of cohort selection. *CGMH* Chang Gung Memorial Hospital, *NCKUH* National Cheng Kung University Hospital, *NTUH* National Taiwan University Hospital, *oGLDs* other glucose-lowering drugs, *SGLT-2is* sodium glucose cotransporter-2 inhibitors, *T1D* type 1 diabetes. *Index date refers to the first date of prescribing SGLT-2is or oGLDs during 2016/05/01–2017/12/31

The selection of study cohort is presented in Fig. 1. The stratification of subgroups by patient baseline eGFR level is detailed in Additional file 1: Figure S2. Between May 2016 and December 2017, there were 11,291, 58,684, and 5973 type 2 diabetes patients who had initiated SGLT-2is or oGLDs from NTUH, CGMH, and NCKUH, respectively, which resulted in a total of 14,020 SGLT-2i and 61,928 oGLD new users (Fig. 1). After 1:1 PS matching, there were 2069, 10,496, and 1101 matched pairs of SGLT-2i and oGLD users, respectively, from NTUH, CGMH, and NCKUH. There were 2300, 5705, and 5509 matched pairs of SGLT-2i and oGLD users in the eGFR ≤ 60, 60 < eGFR ≤ 90, and eGFR > 90 subgroups, respectively (Additional file 1: Figure S2).

Before PS matching, patients in the oGLD group were older, had lower baseline HbA1c and eGFR levels, and were more likely to have established CVDs compared to those in the SGLT-2i group (Additional file 1: Table S1). These differences between SGLT-2i and oGLD users in the overall study cohort are similar to those in the eGFR subgroups, except that the baseline eGFR of oGLD users was slightly higher than that of SGLT-2i users in the eGFR > 90 subgroup (114.7 versus 112.6 mL/min/1.73 m^2^).

After PS matching, all the baseline characteristics between SGLT-2i and oGLD users were comparable, with the standardized differences of most variables being less than 0.1 and those of all variables being less than 0.2 (Table 1). In the overall study population, the mean age at the initiation of SGLT-2is or oGLDs was 60.4 years. Approximately 45% of the patients had microvascular diseases and 37% had a history of CVDs. Among other treatments, statins and angiotensin II receptor blockers were the most frequently prescribed medications in the year before or at the index date. Regarding the three eGFR subgroups, the patients in the eGFR > 90 subgroup were the youngest and had the fewest comorbidities (i.e., average age of 55 years, and 39% and 26% of patients having microvascular diseases and CVDs, respectively).

**Table 1:** Patient characteristics of overall study cohort and subgroups stratified by baseline estimated glomerular filtration rate (eGFR) levels (i.e., ≤ 60, < 60–90, > 90 mL/min/1.73 m^2^) after propensity score matching

Characteristics	Overall		eGFR ≤ 60 mL/min/1.73m^2^		60 < eGFR ≤ 90 mL/min/1.73 m^2^		eGFR > 90 mL/min/1.73 m^2^	
	SGLT-2is n = 13,666	oGLDs n = 13,666	SGLT-2is n = 2300	oGLDs n = 2300	SGLT-2is n = 5705	oGLDs n = 5705	SGLT-2is n = 5509	oGLDs n = 5509
Age (years), mean (SD)	60.4 (11.6)	60.4 (12.5)	67.7 (10.2)	67.6 (11.0)	62.2 (10.3)	62.5 (11.0)	55.6 (11.4)	55.7 (12.1)
Male, n (%)	7922 (58.0)	7803 (57.1)	1312 (57.0)	1319(57.4)	3587 (62.9)	3513 (61.6)	2919 (53.0)	2837 (51.5)
Baseline HbA1c (%), mean (SD)	8.68 (1.5)	8.74 (1.5)	8.7 (1.5)	8.64 (1.6)	8.56 (1.4)	8.61 (1.5)	8.81 (1.5)	8.86 (1.5)
Baseline HbA1c (mmol/mol), mean	71	72	72	71	70	71	73	73
Baseline eGFR (mL/min/1.73 m^2^), mean (SD)	86.03 (27.2)	85.85 (31.6)	48.74 (9.5)	48.5 (10.0)	75.58 (8.5)	75.49 (8.6)	112.56 (19.4)	113.58 (21.2)
eGFR > 90, n (%)	5547 (16.9)	5592 (17.7)	–	–	–	–	5509 (100.0)	5509 (100.0)
60 < eGFR ≤ 90, n (%)	5811 (42.5)	5654 (41.4)	–	–	5705 (100.0)	5705 (100.0)	–	–
eGFR ≤ 60, n (%)	2308 (40.6)	2420 (40.9)	2300 (100.0)	2300 (100.0)	–	–	–	–
eGFR change in the year before the index date (SD) (mL/min/1.73 m^2^)	− 1.59 (13.1)	− 1.55 (15.1)	− 4.13 (9.4)	− 4.26 (11.2)	− 3.02 (11.6)	− 2.92 (13.0)	0.94 (15.6)	0.77 (17.0)
Presence of microvascular diseases, n (%)	6124 (44.8)	6084 (44.5)	1317 (57.3)	1333 (58.0)	2551 (44.7)	2520 (44.2)	2185 (39.7)	2157 (39.2)
History of cardiovascular disease, n (%)								
Myocardial infarction	712 (5.2)	705 (5.2)	177 (7.7)	181 (7.9)	300 (5.8)	327 (5.7)	203 (3.7)	180 (3.3)
Unstable angina	2115 (15.5)	2096 (15.3)	473 (20.6)	465 (20.2)	984 (17.2)	924 (16.2)	645 (11.7)	632 (11.5)
Stroke	1912 (14.0)	1950 (14.3)	485 (21.1)	487 (21.2)	906 (15.9)	937 (16.4)	511 (9.3)	506 (9.2)
Heart failure	1377 (10.1)	1419 (10.4)	430 (18.7)	415 (18.0)	584 (10.2)	575 (10.1)	361 (6.6)	358 (6.5)
Atrial fibrillation	578 (4.2)	585 (4.3)	190 (8.3)	201 (8.7)	269 (4.7)	274 (4.8)	112 (2.0)	107 (1.9)
Peripheral artery disease	683 (5.0)	679 (5.0)	222 (9.7)	217 (9.4)	292 (5.1)	293 (5.1)	160 (2.9)	160 (2.9)
History of frailty, n (%)	1775 (13.0)	1850 (13.5)	423 (18.4)	444 (19.3)	733 (12.8)	776 (13.6)	609 (11.1)	636 (11.5)
History of GLD use, n (%)								
Metformin	10,749 (78.7)	10,657 (78.0)	1540 (67.0)	1523 (66.2)	4548 (79.7)	4492 (78.7)	4513 (81.9)	4489 (81.5)
Sulfonylurea	6355 (46.5)	6062 (44.4)	1246 (54.2)	1192 (51.8)	2585 (45.3)	2500 (43.5)	2407 (43.7)	2250 (40.8)
DPP-4 inhibitor	9670 (70.8)	9461 (69.2)	1715 (74.6)	1706 (74.2)	4083 (71.7)	4024 (70.5)	3731 (67.7)	3613 (65.6)
Thiazolidinedione	3268 (23.9)	2770 (20.3)	559 (24.3)	538 (23.4)	1414 (24.8)	1171 (20.5)	1210 (22.0)	1008 (18.3)
GLP-1 receptor agonist	369 (2.7)	403 (2.9)	54 (2.3)	63 (2.7)	111 (1.9)	112 (2.0)	203 (3.7)	224 (4.1)
Insulin	3054 (22.3)	3200 (23.4)	651 (28.3)	691 (30.0)	1196 (21.0)	1215 (21.3)	1162 (21.1)	1191 (21.6)
Total number of GLD class	2.38 (1.09)	2.45 (1.08)	2.48 (1.11)	2.51 (1.05)	2.37 (1.08)	2.44 (1.08)	2.32 (1.07)	2.40 (1.08)
Other medications, n (%)								
Antihypertensive drug	10,088 (73.8)	10,097 (73.9)	2079 (90.4)	2093 (91.0)	4491 (78.7)	4525 (79.3)	3392 (61.6)	3398 (61.7)
ACE inhibitor	1142 (8.4)	1102 (8.1)	236 (10.3)	231 (10.0)	528 (9.3)	524 (9.2)	361 (6.6)	324 (5.9)
ARB	7829 (57.3)	7830 (57.3)	1663 (72.3)	1695 (73.7)	3531 (61.9)	3538 (62.0)	2537 (46.1)	2528 (45.9)
β-blocker	5021 (36.7)	5007 (36.6)	1184 (51.5)	1176 (51.1)	2274 (39.9)	2244 (39.3)	1484 (26.9)	1501 (27.2)
Loop diuretic	1096 (8.0)	1107 (8.1)	462 (20.1)	486 (21.1)	397 (7.0)	402 (7.0)	230 (4.2)	229 (4.2)
Thiazide diuretic	468 (3.4)	458 (3.4)	123 (5.3)	132 (5.7)	221 (3.9)	220 (3.9)	113 (2.1)	104 (1.9)
Aldosterone antagonist	585 (4.3)	589 (4.3)	210 (9.1)	204 (8.9)	242 (4.2)	248 (4.3)	129 (2.3)	124 (2.3)
Statin	8518 (62.3)	8476 (62.0)	1487 (64.7)	1504 (65.4)	3572 (62.6)	3559 (62.4)	3347 (60.8)	3311 (60.1)

*ARB* angiotensin receptor blocker, DPP-4 inhibitor: dipeptidyl peptidase 4 inhibitor, *eGFR* estimated glomerular filtration rate, *GLD* glucose-lowering drug, *GLP-1 receptor agonist* glucagon-like peptide 1 receptor agonist, *HbA1c* hemoglobin A1c, *oGLDs* other glucose-lowering drugs, *SD* standard deviation, *SE* standard error, *SGLT-2is* sodium glucose cotransporter-2 inhibitors

The composition of follow-up time of individual SGLT-2is and oGLDs in the overall study cohort and stratified by the three healthcare delivery systems is shown in Additional file 1: Table S2. In the SGLT-2i group, the follow-up time of empagliflozin was slightly longer than that of dapagliflozin (i.e., 52.8% versus 47.2%). In the oGLD group, the percentage of follow-up time was 16.8% for sulfonylureas, 16.3% for DPP-4 inhibitors, 15.1% for metformin, and 14.6% for thiazolidinediones. The mean time from SGLT-2i initiation to the end of follow-up for 30%, 40%, and 50% eGFR reduction was 9.17, 9.20, and 9.20 months, respectively, and that from oGLD initiation to the end of follow-up for 30%, 40%, and 50% eGFR reduction was 8.10, 8.14, and 8.16 months, respectively (Additional file 1: Table S3).

**Fig. 2:**
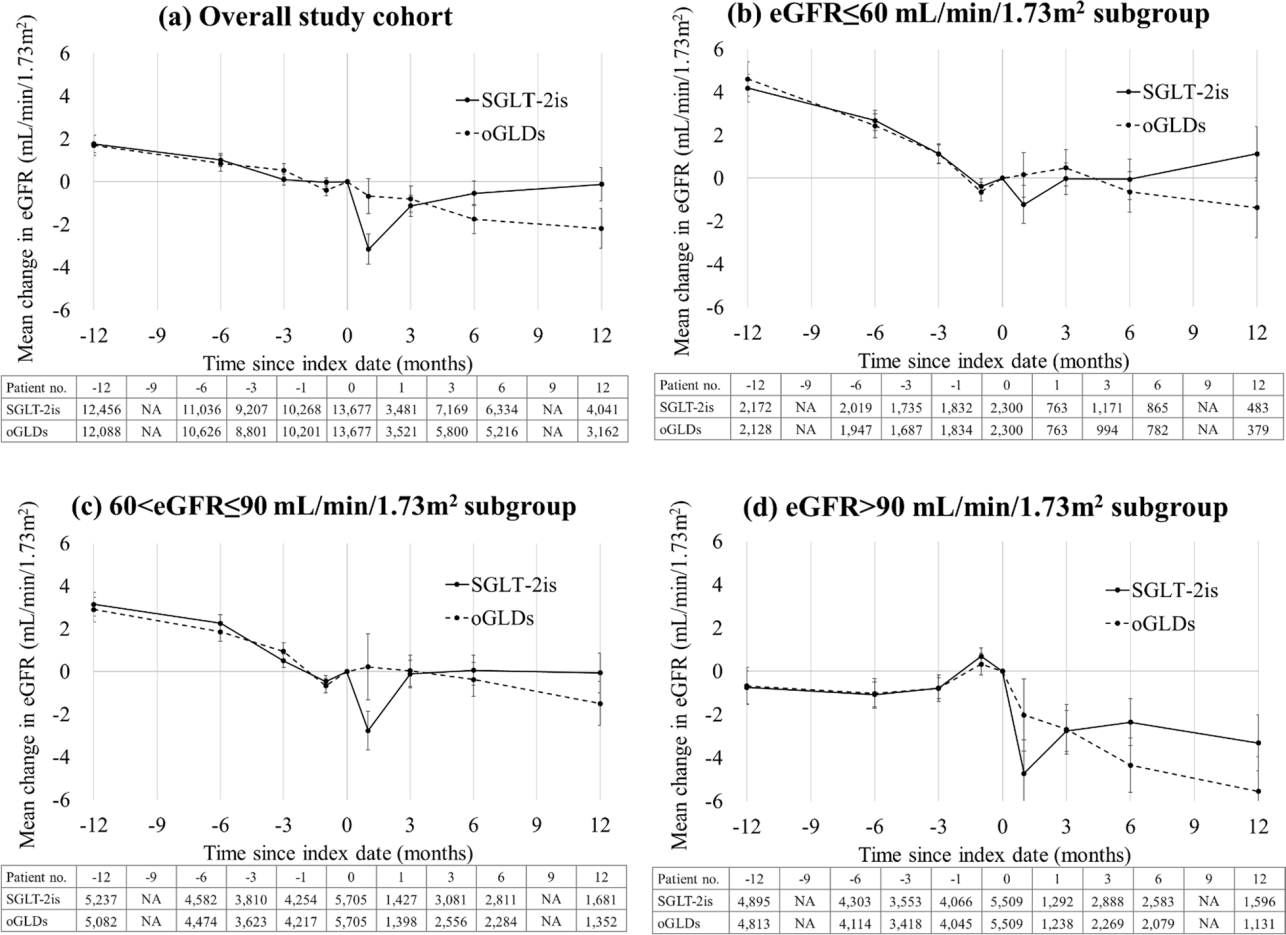
Change in estimated glomerular filtration rate (eGFR) over time before and after index date (i.e., initiation of SGLT-2i or oGLD therapy) (on-treatment analysis): **a** overall study cohort, **b** eGFR ≤ 60 mL/min/1.73 m^2^ subgroup, **c** 60 < eGFR ≤ 90 mL/min/1.73 m^2^ subgroup, and **d** eGFR > 90 mL/min/1.73 m^2^) subgroup. *oGLDs* other glucose-lowering drugs, *SGLT-2is* sodium glucose cotransporter-2 inhibitors. Means of change in eGFR are plotted with standard error bars. The bottom tables present the number of eGFR observations available at each time point

As shown in Fig. 2, in the overall study cohort, the eGFR values of oGLD users showed a stable decline (mean values [standard errors] from 85.6 [0.43] to 82.5 [0.44] mL/min/1.73 m^2^ during the 12 months after treatment initiation), whereas those of SGLT-2i users decreased in the first 3 months of treatment (85.7 [0.37] to 79.7 [0.41] mL/min/1.73 m^2^) but then improved with an average monthly increase of 0.84 mL/min/1.73 m^2^ in the last 9 months. Although the patterns of these changes between the two drug groups were slightly different across the three eGFR subgroups, in general, the use of SGLT-2is attenuated the decline of eGFR levels in all subgroups. Consistent patterns of eGFR changes between the two drug groups were shown in the individual healthcare delivery systems (Additional file 1: Figure S3).

**Fig. 3:**
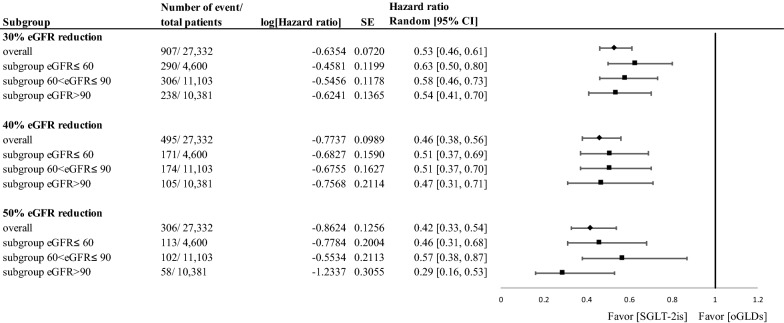
Forest plots for 30%, 40%, and 50% estimated glomerular filtration rate (eGFR) reduction of SGLT-2is versus oGLDs in overall study cohort and subgroups stratified by baseline eGFR level (i.e., ≤ 60, < 60–90, > 90 mL/min/1.73 m^2^) (intention-to-treat analysis). *CI* confidence interval, *oGLDs* other glucose-lowering drugs, *SE* standard error, *SGLT-2is* sodium glucose cotransporter-2 inhibitors

The results of Cox regression analysis with the ITT approach show that the initiation of SGLT-2is was significantly associated with slower eGFR declines (Fig. 3), with HRs (95% CIs) of 0.53 (0.46–0.61), 0.46 (0.38–0.56), and 0.42 (0.33–0.54) for 30%, 40%, and 50% eGFR reduction, respectively. The eGFR change of SGLT-2i versus oGLD use seems more profound in the eGFR > 90 subgroup (in terms of lower HR values) but with wider CIs. Consistent results were found in the analysis with the OT approach (Additional file 1: Figure S4) and from the individual healthcare delivery systems (Additional file 1: Figures S5 and S6).

## Discussion

This is the largest real-world study (more than 25,000 patients) of an Asian population to examine the renoprotective effect of SGLT-2i in a cohort of type 2 diabetes patients and patient subgroups with different baseline kidney functions. A sizable number of type 2 diabetes patients from three large healthcare delivery systems in Taiwan ensured that study results could be applicable to Asian settings. Also, given a universal healthcare coverage in Taiwan, the results based on the data from Taiwan’s setting would avoid potential confounding bias attributable to patient socioeconomic status. The benefit of SGLT-2i in preserving kidney function for type 2 diabetes patients was consistent across patient groups at different baseline eGFR levels.

Our findings are comparable to those from existing trials [1, 3, 5, 23, 24] and real-world evidence [13] from other countries or ethnic populations, thereby supporting that similar favorable kidney outcomes with SGLT-2i use are observed in an Asian population. Among existing evidence, CVD-REAL 3 [13], which produced the first and currently the largest real-world data on the effects of SGLT2i therapy on kidney outcomes, comprised patients with an average baseline eGFR level of 91 mL/min/1.73 m^2^. Less than 10% of patients had baseline eGFR levels of ≤ 60 mL/min/1.73 m^2^. Compared to CVD-REAL 3 [13], our study patients had poorer baseline kidney function. The average eGFR level in this study was 85 mL/min/1.73 m^2^ and 40% of patients had a baseline eGFR level of ≤ 60 mL/min/1.73 m^2^. Moreover, the prevalence of prior CVDs (including atherosclerotic CVDs and heart failure) in this study was higher than that of CVD-REAL 3 (37% vs. 22%). The composition of our study cohort is more similar to that of patients enrolled in the DECLARE-TIMI 58 trial [3], where patients had a mean eGFR value of 85.2 mL/min/1.73 m^2^ and a slightly higher proportion (40.6%) had atherosclerotic CVDs. It is noteworthy that despite the differences in patient characteristics and study designs across studies, the benefit of restoring kidney function associated with SGLT-2i versus oGLD use was similar; i.e., HR (95% CI) for 40% eGFR reduction was 0.46 (0.38–0.56) in our study, 0.56 (0.45–0.70) in CVD-REAL 3 [13], and 0.54 (0.43–0.67) in the DECLARE-TIMI 58 trial [3]. Our results suggest that the evidence on the renoprotective effect of SGLT-2i therapy from existing trials and real-world studies can be extended to a large Asian population.

In our study, the renoprotective effect of SGLT-2i use was consistently observed across the eGFR subgroups, while the pattern of eGFR change was slightly different among the subgroups (Figs. 2 and 3). However, interpretation of the results should be done with caution considering that the mean duration of follow-up was only 8–9 months and that not all the patients had eGFR data available from each study time point during in follow-up. To the best of our knowledge, only a few previous studies examined the kidney outcomes of SGLT-2i use for patients with different baseline kidney function. In the CREDENCE trial [5], the beneficial effect of canagliflozin on kidney outcomes was found to be consistent across the eGFR subgroups, as determined by testing the interaction between the treatment status (i.e., canagliflozin versus placebo) and subgroups (i.e., eGFR subgroups). However, this trial only included type 2 diabetes patients who had albuminuric CKD with eGFR of below 90 mL/min/1.73 m^2^. Our study results further support that the renoprotective effect of SGLT-2i use is consistent across different eGFR subgroups, even in patients without CKD. Although the eGFR changes in the subgroups of eGFR ≤ 60, 60 to ≤ 90, and > 90 mL/min/1.73 m^2^ were analyzed in the CVD-REAL 3 study [13], the trajectory of eGFR changes over time in each subgroup was not reported. Additionally, in the CVD-REAL 3 study, PS matching was only conducted for the overall study cohort, so the subgroup analysis was simply performed after PS matching with further adjustment for important covariates in the Cox models. We improved the internal validity of the study findings by also performing PS matching within each eGFR subgroup.

Lin et al. [15] conducted a retrospective study of 7624 matched pairs of SGLT-2i and SGLT-2i non-users based on the CGRD from May 2016 to December 2017. They showed that SGLT-2i use significantly lowered the risk of eGFR reduction by over 40% and acute kidney injury (AKI)-related hospitalization in the year after SGLT-2i initiation among the patient subgroups with baseline eGFR levels of ≥ 90 and 60–89 mL/min/1.73 m^2^; the reduction was less prominent among those with baseline eGFR values of 59 mL/min/1.73 m^2^ or lower. Lin et al.’s study included patients from only one healthcare system (i.e., CGMH network) and had fewer patients compared to the present study. PS matching between SGLT-2i and non-users was performed in Lin et al.’s study [15], but the baseline eGFR between treatment groups remained significantly different (i.e., lower eGFR level for SGLT-2i users versus non-users). In the present study, we carefully identified eGFR subgroups with sufficient numbers of patients (Additional file 1: Figure S2) and conducted detailed matching procedures within each subgroup to achieve greater level of comparability in baseline patient characteristics between treatment users in each subgroup. These analytic procedures ensure the validity of our results.

A check-mark-shaped eGFR change (or called “eGFR dip”) following SGLT-2i initiation has been consistently observed in previous trials [1, 3, 5, 23, 24]. An initial drop in eGFR levels followed by an increase and subsequent stabilization was observed in the present study. This biphasic eGFR change after SGLT-2i initiation suggests reduced hyperfiltration in viable nephrons at the beginning of SGLT2i therapy that translates into the preservation of kidney function. Current evidence shows that the transient decrease in eGFR, while is more likely to be observed in patients with more advanced kidney diseases and diuretic therapy, does not impact treatment response or subsequent cardiovascular and kidney outcomes [25]. Only in rare cases with an initial eGFR dip > 30%, SGLT-2i treatment should be temporarily held until eGFR level returns to baseline.

Intrarenal hemodynamic mechanisms of nephroprotection by SGLT-2 inhibition have been proposed, where the reduced reabsorption of sodium in the proximal tubule by SGLT-2i could be essential for alleviating glomerular hypertension and hyperfiltration [26]. The use of SGLT-2is can reduce these unwanted effects by restoring normal tubuloglomerular feedback activity and eGFR, thereby reducing injury to glomeruli and tubulointerstitium. Moreover, this renoprotective effect seems to be a class effect of SGLT-2is, as supported by the consistent biphasic eGFR change observed in patients treated with different individual SGLT-2is [26]. In the present study, the pattern of eGFR response to SGLT-2i use was consistent across patient groups with different baseline eGFR levels, further suggesting that this effect may be independent of the severity of kidney damage of type 2 diabetes patients.

The present study has a few limitations. First, like other retrospective studies, biases due to residual confounding may not have been excluded. However, this concern was minimized because we included for all measurable confounding factors and carefully identified potential proxies for intangible variables (e.g., frailty), and adjusted both in the analysis. Second, we only focused on the short-term kidney effect of SGLT-2is; the long-term hard outcomes (e.g., kidney failure with or without replacement therapy) were not measured and analyzed. Because most patients in the present study were observed less than one year after the initiation of the study drugs, real-world studies with a longer follow-up period are needed to examine the long-term kidney effect of SGLT-2i use. Third, although the baseline HbA1c have been adjusted in the matching procedures, data on glycemic control such as HbA1c during follow-up were not measured considering that our main interest focused on kidney outcomes after treatment initiation. Also, urinary albumin/creatinine ratios (UACRs) at baseline or follow-up were not collected due to data incompleteness. Not all study patients were proteinuria or had CKD and thus routine check-up data on UACR may not be available. Even in patients with CKD, the rate of UACR testing in clinical settings has been reported to be low or highly variable [27, 28]. Fourth, we did not analyze individual SGLT-2is or explore the dose–response effect of SGLT-2i therapy. Fifth, only the clinical effectiveness of SGLT-2is was studied; we did not examine the safety profiles of treatment. For example, SGLT-2i-related AKI is of concern, especially among patients with poor kidney function. However, Lin et al.’s study of Taiwanese patients with type 2 diabetes showed that the use of SGLT-2is was not associated with an increased risk of AKI-related hospitalization across patients with different baseline kidney function [15]. Sixth, the generic inverse-variance method with random effects in the meta-analysis was adopted to generate more conservative aggregated estimates from the results obtained from three study healthcare systems. However, it remains uncertain if heterogeneity in clinical practice and patient composition across different healthcare systems can be completely handled in the random effects model approach considering that not all differences across the healthcare systems were measured in the analysis. Lastly, the interpretation of our results may be limited to a Taiwanese or Asian population with type 2 diabetes under a healthcare setting with universal health insurance coverage. Nevertheless, because regular checkups (i.e., monitoring of patient kidney function every six months to one year) are reimbursed in such settings, clinical practice is likely to adhere to clinical guidelines and patient laboratory data associated with patient kidney function are thus more complete, which ensures data quality.

## Conclusions

The use of SGLT-2is versus oGLDs yielded a favorable effect in slowing eGFR declines, regardless of patient baseline renal function, in a real-world type 2 diabetes population. Our results extend the evidence from existing trials and real-world studies on the renoprotective effect of SGLT-2i to a large Asian population.

## Supplementary Information

**Additional file 1:** **Table S1.** Patient characteristics of overall study cohort and subgroups stratified by baseline estimated glomerular filtration rate (eGFR) level (i.e., ≤60, <60-90, >90 mL/min/1.73 m^2^) before propensity score matching. **Table S2.** Composition of sodium glucose cotransporter-2 inhibitor (SGLT-2i) and other glucose-lowering drug (oGLD) groups in terms of total follow-up time in overall cohort. **Table S3.** Mean follow-up time (months) for sodium glucose cotransporter-2 inhibitor (SGLT-2i) and other glucose-lowering drug (oGLD) (intention-to-treat analysis). **Figure S1.** Data elements of the study-specific common data model. **Figure S2.** Flow chart of cohort selection. **Figure S3.** Change in estimated glomerular filtration rate (eGFR) over time before and after initiation of sodium glucose cotransporter-2 inhibitor (SGLT-2i) or other glucose-lowering drug (oGLD) therapy (on-treatment analysis) in overall study cohort from (a) Chang Gung Memorial Hospital (CGMH), (b) National Cheng Kung University Hospital (NCKUH), and (c) National Taiwan University Hospital (NTUH). **Figure S4. **Forest plots for 30%, 40%, and 50% eGFR reduction of sodium glucose cotransporter-2 inhibitor (SGLT-2i) versus other glucose-lowering drug (oGLD) use in overall study cohort and in each estimated glomerular filtration rate (eGFR) subgroup (on-treatment analysis). **Figure S4.** Forest plots for 30%, 40%, and 50% eGFR reduction of sodium glucose cotransporter-2 inhibitor (SGLT-2i) versus other glucose-lowering drug (oGLD) use in overall study cohort and in each estimated glomerular filtration rate (eGFR) subgroup (on-treatment analysis). **Figure S5.** Forest plots for 30%, 40%, and 50% estimated glomerular filtration rate (eGFR) reduction of sodium glucose cotransporter-2 inhibitor (SGLT-2i) versus other glucose-lowering drug (oGLD) use (intention-to-treat analysis) in overall study cohort from CGMH, NCKUH, and NTUH. **Figure S6.** Forest plots for 30%, 40%, and 50% estimated glomerular filtration rate (eGFR) reduction of sodium glucose cotransporter-2 inhibitor (SGLT-2i) versus other glucose-lowering drug (oGLD) use (on-treatment analysis) in overall study cohort from CGMH, NCKUH, and NTUH.

## Data Availability

All data generated or analyzed are included in this article and its additional information files.

## Abbreviations

AKI: Acute kidney injury
CDM: Common data model
CGRD: Chang Gung Research Database
CGMH: Chang Gung Memorial Hospital
CIs: Confidence intervals
CKD: Chronic kidney disease
CVD-REAL 3: Comparative Effectiveness of Cardiovascular Outcomes in New Users of SGLT2 Inhibitors 3
CVDs: Cardiovascular diseases
CVOTs: Cardiovascular outcome trials
DPP-4: Dipeptidyl peptidase-4
eGFR: Estimated glomerular filtration rate
EHRs: Electronic health records
GLP-1: Glucagon-like peptide-1
HbA1c: Hemoglobin A1c
HRs: Hazard ratios
ITT: Intention-to-treat
NCKUH: National Cheng Kung University Hospital
NHI: National Health Insurance
NTUH: National Taiwan University Hospital
oGLDs: Other glucose-lowering drugs
OT: On-treatment
PS: Propensity score
SCr: Serum creatinine
SGLT-2is: Sodium glucose cotransporter-2 inhibitors
