# Supplementary material for: Renoprotective effect of SGLT-2 inhibitors among type 2 diabetes patients with different baseline kidney function: a multi-center study
Source: Cardiovasc Diabetol. 2021 Oct 7;20:203. doi: 10.1186/s12933-021-01396-2 (PMC8499571; doi:10.1186/s12933-021-01396-2)
Supplement: Supplementary file 1 — Additional file 1: Table S1. Patient characteristics of overall study cohort and subgroups stratified by baseline estimated glomerular filtration rate (eGFR) level (i.e., ≤60, <60-90, >90 mL/min/1.73 m2) before propensity score matching. Table S2. Composition of sodium glucose cotransporter-2 inhibitor (SGLT-2i) and other glucose-lowering drug (oGLD) groups in terms of total follow-up time in overall cohort. Table S3. Mean follow-up time (months) for sodium glucose cotransporter-2 inhibitor (SGLT-2i) and other glucose-lowering drug (oGLD) (intention-to-treat analysis). Figure S1. Data elements of the study-specific common data model. Figure S2. Flow chart of cohort selection. Figure S3. Change in estimated glomerular filtration rate (eGFR) over time before and after initiation of sodium glucose cotransporter-2 inhibitor (SGLT-2i) or other glucose-lowering drug (oGLD) therapy (on-treatment analysis) in overall study cohort from (a) Chang Gung Memorial Hospital (CGMH), (b) National Cheng Kung University Hospital (NCKUH), and (c) National Taiwan University Hospital (NTUH). Figure S4. Forest plots for 30%, 40%, and 50% eGFR reduction of sodium glucose cotransporter-2 inhibitor (SGLT-2i) versus other glucose-lowering drug (oGLD) use in overall study cohort and in each estimated glomerular filtration rate (eGFR) subgroup (on-treatment analysis). Figure S4. Forest plots for 30%, 40%, and 50% eGFR reduction of sodium glucose cotransporter-2 inhibitor (SGLT-2i) versus other glucose-lowering drug (oGLD) use in overall study cohort and in each estimated glomerular filtration rate (eGFR) subgroup (on-treatment analysis). Figure S5. Forest plots for 30%, 40%, and 50% estimated glomerular filtration rate (eGFR) reduction of sodium glucose cotransporter-2 inhibitor (SGLT-2i) versus other glucose-lowering drug (oGLD) use (intention-to-treat analysis) in overall study cohort from CGMH, NCKUH, and NTUH. Figure S6. Forest plots for 30%, 40%, and 50% estimated glomerular filtration rat [file 12933_2021_1396_MOESM1_ESM.pdf]

SUPPLEMENTARY INFORMATION

ARTICLE TITLE: Renoprotective Effect of SGLT-2 Inhibitors among Type 2 Diabetes Patients with Different Baseline Kidney Function: A Multi-Center Study

AUTHORS: Fang-Ju Lin, PhD; hi-Chuan Wang, PhD; Chien-Ning Hsu, PhD, RPh; Chen-Yi Yang, MS; Chih-Yuan Wang, MD, PhD; Huang-Tz Ou, PhD

Table S1. Patient characteristics of overall study cohort and subgroups stratified by baseline estimated glomerular filtration rate (eGFR) level (i.e., ≤60, <60-90, >90 mL/min/1.73 m^2) before propensity score matching

Characteristics	Overall		eGFR≤60 mL/min/1.73 m^2		60<eGFR≤90 mL/min/1.73 m^2		eGFR>90 mL/min/1.73 m^2	
	SGLT-2is n=14,020	oGLDs n=61,928	SGLT-2is n=2,323	oGLDs n=23,295	SGLT-2is n=5,959	oGLDs n=20,301	SGLT-2is n=5,738	oGLDs n=18,332
Age (years), mean (SD)	60.3 (11.6)	65.5 (13.0)	67.7 (10.2)	70.8 (11.4)	62.1 (10.3)	65.5 (11.6)	55.5 (11.4)	58.8 (13.1)
Male, n (%)	8,169 (58.3)	32,970 (53.2)	1,331 (57.3)	12,219 (52.5)	3,780 (63.4)	11,919 (58.7)	3,058 (53.3)	9,388 (51.2)
Baseline HbA1c (%), mean (SD)	8.68 (1.5)	8.33 (1.7)	8.66 (1.5)	8.23 (1.8)	8.54 (1.4)	8.28 (1.6)	8.81 (1.5)	8.52 (1.7)
Baseline HbA1c (mmol/mol), mean*	71	68	71	66	70	67	73	70
Baseline eGFR (mL/min/1.73 m^2), mean (SD)	86.3 (27.3)	72.0 (36.3)	48.8 (9.5)	35.8 (16.6)	75.6 (8.48)	75.1 (8.56)	112.6 (19.4)	114.7 (23.1)
eGFR>90, n (%)	5,738 (40.9)	18,332 (29.6)	-	-	-	-	5,738 (100.0)	18,332 (100.0)
60<eGFR≤90, n (%)	5,959 (42.5)	20,301 (32.8)	-	-	5,959 (100.0)	20,301 (100.0)	-	-
eGFR≤60, n (%)	2,323 (16.6)	23,295 (37.6)	2,323 (100.0)	23,295 (100.0)	-	-	-	-
eGFR change in year before index date (SD) (mL/min/1.73 m^2)	-1.56 (13.2)	-1.86 (16.1)	-4.12 (9.4)	-5.80 (12.5)	-2.94 (11.7)	-2.55 (14.1)	0.91 (15.3)	3.90 (20.1)
Presence of microvascular diseases, n (%)	6,495 (46.3)	27,118 (43.8)	1,321 (56.9)	13,133 (56.4)	2,666 (44.7)	7,316 (36.0)	2,278 (39.7)	5,691 (31.0)
History of cardiovascular disease, n (%)								
Myocardial infarction	741 (5.3)	3,453 (5.6)	177 (7.6)	1,994 (8.6)	352 (5.9)	976 (4.8)	212 (3.7)	483 (2.6)
Unstable angina	2,120 (15.1)	9,415 (15.2)	477 (20.5)	4,516 (19.4)	1,026 (17.2)	3,120 (15.4)	666 (11.6)	1,887 (10.3)
Stroke	1,926 (13.7)	14,715 (23.8)	486 (20.9)	7,125 (30.6)	924 (15.5)	4,661 (23.0)	516 (9.0)	2,929 (16.0)
Heart failure	1,405 (10.0)	8,847 (14.3)	432 (18.6)	5,457 (23.4)	599 (10.1)	2,221 (10.9)	374 (6.5)	1,169 (6.4)

Atrial fibrillation	594 (4.2)	4,272 (6.9)	191 (8.2)	2,389 (10.3)	285 (4.8)	1,277 (6.3)	116 (2.0)	596 (3.3)
Peripheral artery disease	701 (5.0)	4,774 (7.7)	224 (9.6)	2,895 (12.4)	307 (5.2)	1,168 (5.8)	170 (3.0)	711 (3.9)
History of frailty, n (%)	1,782 (12.7)	21,831 (35.3)	423 (18.2)	10,484 (45.0)	742 (12.5)	5,862 (28.9)	617 (10.8)	5,485 (29.9)
History of GLD use, n (%)								
Metformin	11,098 (79.2)	30,710 (49.6)	1,563 (67.3)	8,495 (36.5)	4,798 (80.5)	11,311 (55.7)	4,737 (82.6)	10,904 (59.5)
Sulfonylurea	6,649 (47.4)	19,804 (32.0)	1,265 (54.5)	8,938 (38.4)	2,792 (46.9)	5,797 (28.6)	2,592 (45.2)	5,069 (27.7)
DPP-4 inhibitor	10,004 (71.4)	27,319 (44.1)	1,737 (74.8)	12,000 (51.5)	4,327 (72.6)	8,206 (40.4)	3,940 (68.7)	7,113 (38.8)
Thiazolidinedione	3,540 (25.2)	5,725 (9.2)	571 (24.6)	2,426 (10.4)	1,597 (26.8)	1,781 (8.8)	1,372 (23.9)	1,518 (8.3)
GLP-1 receptor agonist	381 (2.7)	1,208 (2.0)	54 (2.3)	462 (2.0)	117 (2.0)	296 (1.5)	210 (3.7)	450 (2.5)
Insulin	3,118 (22.2)	14,619 (23.6)	658 (28.3)	7,489 (32.1)	1,246 (20.9)	3,573 (17.6)	1,214 (21.2)	3,557 (19.4)
Total number of GLD class	1.60 (1.16)	2.48 (1.09)	1.71 (1.17)	2.52 (1.05)	1.53 (1.15)	2.50 (1.09)	1.56 (1.16)	2.45 (1.10)
Other medications, n (%)								
Antihypertensive drug	10,376 (74.0)	45,318 (73.2)	2,102 (90.5)	19,832 (85.1)	4,717 (79.2)	14,857 (73.2)	3,557 (62.0)	10,589 (57.8)
ACE inhibitor	1,171 (8.4)	4,923 (7.9)	238 (10.2)	2,204 (9.5)	559 (9.4)	1,636 (8.1)	374 (6.5)	1,083 (5.9)
ARB	8,063 (57.5)	32,140 (51.9)	1,681 (72.4)	14,486 (62.2)	3,711 (62.3)	10,671 (52.6)	2,671 (46.5)	6,984 (38.1)
β-blocker	5,182 (37.0)	22,804 (36.8)	1,199 (51.6)	10,955 (47.0)	2,413 (40.5)	7,135 (35.1)	1,570 (27.4)	4,714 (25.7)
Loop diuretic	1,107 (7.9)	11,768 (19.0)	466 (20.1)	7,830 (33.6)	407 (6.8)	2,325 (11.5)	234 (4.1)	1,613 (8.8)
Thiazide diuretic	492 (3.5)	2,161 (3.5)	128 (5.5)	1,170 (5.0)	240 (4.0)	623 (3.1)	124 (2.2)	368 (2.0)
Aldosterone antagonist	598 (4.3)	4,212 (6.8)	211 (9.1)	2,413 (10.4)	254 (4.3)	1,083 (5.3)	133 (2.3)	716 (3.9)
Statin	8,781 (62.6)	31,150 (50.3)	1,505 (64.8)	12,027 (51.6)	3,755 (62.3)	10,613 (52.3)	3,521 (61.4)	8,510 (46.4)

Abbreviations: ARB: angiotensin receptor blocker; DPP-4 inhibitor: dipeptidyl peptidase 4 inhibitor; GLD: glucose-lowering drug; GLP-1 receptor agonist: glucagon-like peptide 1 receptor agonist; HbA1c: hemoglobin A1c; oGLDs: other glucose-lowering drugs; SD: standard deviation; SE: standard error; SGLT-2is: sodium glucose cotransporter-2 inhibitors.

Table S2. Composition of sodium glucose cotransporter-2 inhibitor (SGLT-2i) and other glucose-lowering drug (oGLD) groups in terms of total follow-up time in overall cohort

Years of follow-up (% of time in overall study cohort and within each study healthcare delivery system)	Overall	CGMH	NCKUH	NTUH
SGLT-2i group				
Dapagliflozin	4950 (47.2)	3,764 (45.8)	162 (25.4)	1,023 (63.2)
Empagliflozin	5528 (52.8)	4,455 (54.2)	476 (74.6)	597 (36.8)
oGLD group				
Metformin	1,404 (15.1)	1,204 (16.7)	27 (4.7)	173 (11.2)
Sulfonylurea	1,566 (16.8)	1,204 (16.7)	105 (18.2)	258 (16.7)
Meglitinides	257 (2.8)	144 (2.0)	42 (7.3)	72 (4.6)
DPP-4 inhibitor	1,519 (16.3)	1,092 (15.2)	121 (21.1)	306 (19.8)
Thiazolidinedione	1,355 (14.6)	1,043 (14.5)	63 (11.0)	249 (16.2)
Acarbose	1,246 (13.4)	977 (13.6)	80 (13.9)	189 (12.3)
GLP-1 receptor agonist	588 (6.3)	487 (6.8)	21 (3.6)	80 (5.2)
Insulin	1,376 (14.8)	1,046 (14.5)	115 (20.1)	215 (13.9)

Abbreviations: CGMH: Chang Gung Memorial Hospital; DPP-4 inhibitor: dipeptidyl peptidase 4 inhibitor; GLP-1 receptor agonist: glucagon-like peptide 1 receptor agonist; NCKUH: National Cheng Kung University Hospital; NTUH: National Taiwan University Hospital.

Table S3. Mean follow-up time (months) for sodium glucose cotransporter-2 inhibitor (SGLT-2i) and other glucose-lowering drug (oGLD) (intention-to-treat analysis)

	Overall (n=27,332)	CGMH (n=20,992)	NCKUH (n=2,202)	NTUH (n=4,138)
30% eGFR reduction				
SGLT-2i group	9.17	9.38	6.96	9.28
oGLD group	8.10	8.16	6.19	8.77
40% eGFR reduction				
SGLT-2i group	9.20	9.40	6.96	9.35
oGLD group	8.14	8.20	6.22	8.83
50% eGFR reduction				
SGLT-2i group	9.20	9.40	6.96	9.37
oGLD group	8.16	8.22	6.23	8.88

Abbreviations: CGMH: Chang Gung Memorial Hospital; eGFR: estimated glomerular filtration rate; NCKUH: National Cheng Kung University Hospital; NTUH: National Taiwan University Hospital.

Figure S1. Data elements of the study-specific common data model

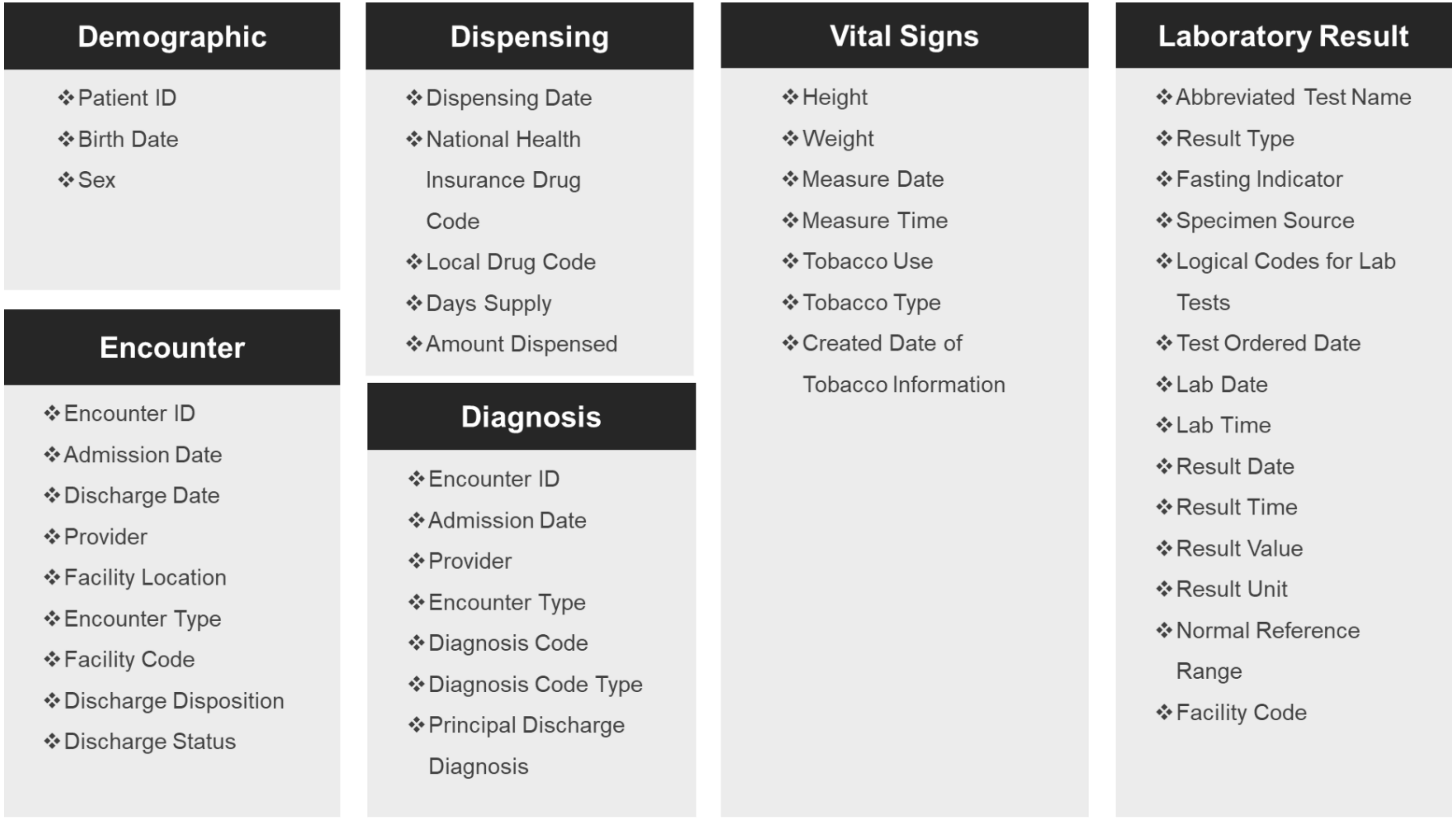

Figure S2. Flow chart of cohort selection

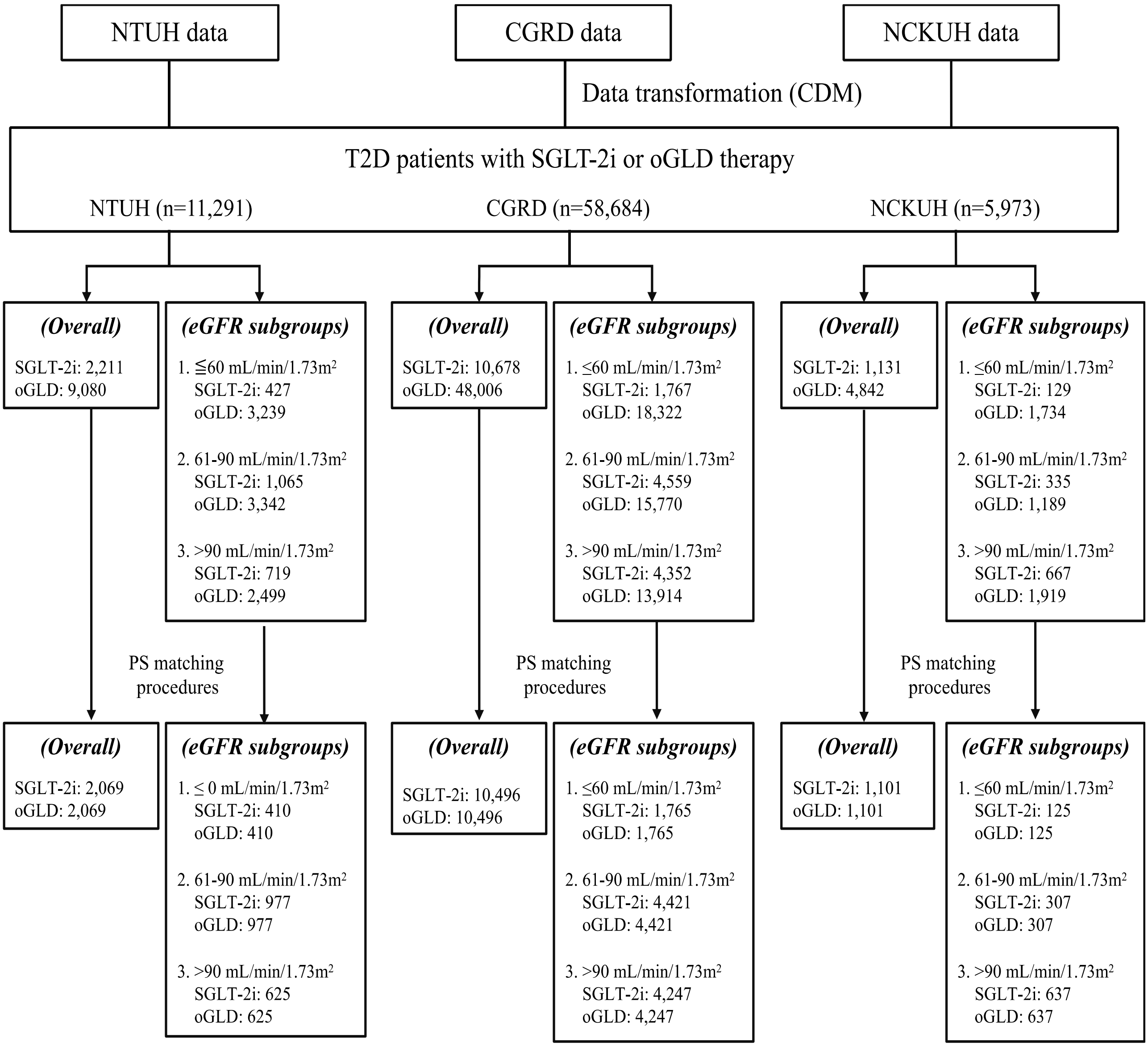

Abbreviations: CGMH: Chang Gung Memorial Hospital; CDM: common data model; eGFR: estimated glomerular filtration rate; NCKUH: National Cheng Kung University Hospital; NTUH: National Taiwan University Hospital; oGLD: other glucose-lowering drug; PS: propensity score; SGLT-2i: sodium glucose cotransporter-2 inhibitor.

Note: PS matching procedures were conducted for matching SGLT-2i users with those treated with oGLDs.

Figure S3. Change in estimated glomerular filtration rate (eGFR) over time before and after initiation of sodium glucose cotransporter-2 inhibitor (SGLT-2i) or other glucose-lowering drug (oGLD) therapy (on-treatment analysis) in overall study cohort from (a) Chang Gung Memorial Hospital (CGMH), (b) National Cheng Kung University Hospital (NCKUH), and (c) National Taiwan University Hospital (NTUH)

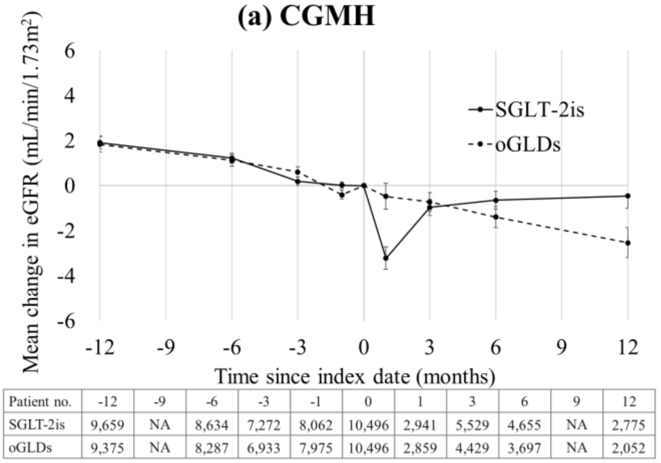

Patient no.	-12	-9	-6	-3	-1	0	1	3	6	9	12
SGLT-2is	9,659	NA	8,634	7,272	8,062	10,496	2,941	5,529	4,655	NA	2,775
oGLDs	9,375	NA	8,287	6,933	7,975	10,496	2,859	4,429	3,697	NA	2,052

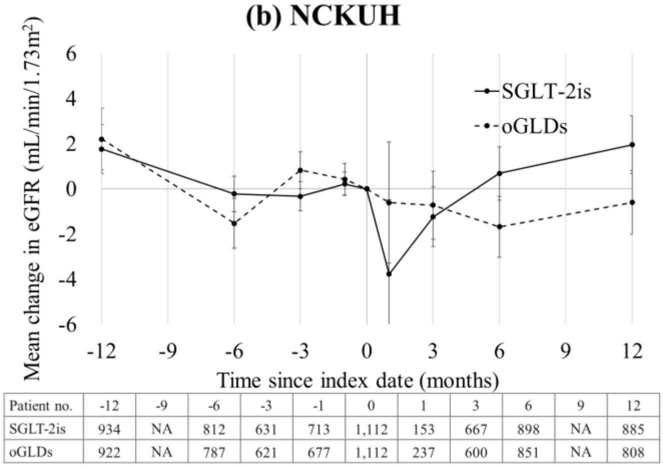

Patient no.	-12	-9	-6	-3	-1	0	1	3	6	9	12
SGLT-2is	934	NA	812	631	713	1,112	153	667	898	NA	885
oGLDs	922	NA	787	621	677	1,112	237	600	851	NA	808

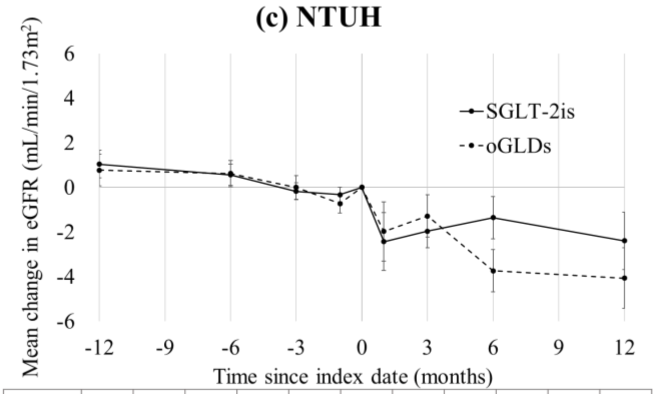

Patient no.	-12	-9	-6	-3	-1	0	1	3	6	9	12
SGLT-2is	1,872	NA	1,590	1,304	1,493	2,069	387	973	781	NA	381
oGLDs	1,791	NA	1,552	1,247	1,549	2,069	425	771	668	NA	302

Note: Means of change in eGFR are plotted with standard error bars. The bottom tables present the number of eGFR observations available at each time point.

Figure S4. Forest plots for 30%, 40%, and 50% eGFR reduction of sodium glucose cotransporter-2 inhibitor (SGLT-2i) versus other glucose-lowering drug (oGLD) use in overall study cohort and in each estimated glomerular filtration rate (eGFR) subgroup (on-treatment analysis)

Subgroup	Number of event/ total patients	log[Hazard ratio]	SE	Hazard ratio Random [95% CI]
**30% eGFR reduction**				
overall	288/ 27,332	-0.8060	0.1244	0.45 [0.35, 0.57]
subgroup eGFR≤60	83/ 4,600	-0.4238	0.2264	0.65 [0.42, 1.02]
subgroup 60<eGFR≤90	77/ 11,103	-0.5124	0.2459	0.60 [0.37, 0.97]
subgroup eGFR>90	97/ 10,381	-0.8192	0.2136	0.44 [0.29, 0.67]
**40% eGFR reduction**				
overall	129/ 27,332	-1.4457	0.2303	0.24 [0.15, 0.37]
subgroup eGFR≤60	34/ 4,600	-0.8630	0.3808	0.42 [0.20, 0.89]
subgroup 60<eGFR≤90	37/ 11,103	-0.9816	0.3741	0.37 [0.18, 0.78]
subgroup eGFR>90	29/ 10,381	-0.5265	1.7024	0.59 [0.02, 16.61]
**50% eGFR reduction**				
overall	70/ 27,332	-1.7583	0.3315	0.17 [0.09, 0.33]
subgroup eGFR≤60	15/ 4,600	-1.3935	0.6458	0.25 [0.07, 0.88]
subgroup 60<eGFR≤90	23/ 11,103	-0.8236	0.4547	0.44 [0.18, 1.07]
subgroup eGFR>90	14/ 10,381	-2.6492	0.9980	0.07 [0.01, 0.50]

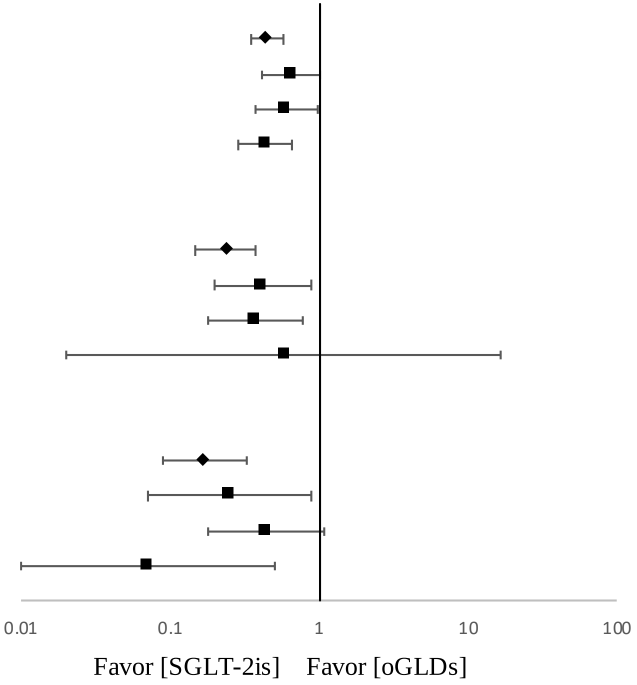

Abbreviation: SE: standard error, CI: confidence interval.

Figure S5. Forest plots for 30%, 40%, and 50% estimated glomerular filtration rate (eGFR) reduction of sodium glucose cotransporter-2 inhibitor (SGLT-2i) versus other glucose-lowering drug (oGLD) use (intention-to-treat analysis) in overall study cohort from CGMH, NCKUH, and NTUH

Subgroup	Number of event/ total patients	log[Hazard ratio]	SE	Weight	Hazard ratio Random [95% CI]
**30% eGFR reduction**					
CGMH	744/ 20,992	-0.6464	0.0776	81.7%	0.52 [0.45, 0.61]
NCKUH	43/ 2,202	-0.8280	0.3274	4.5%	0.44 [0.23, 0.83]
NTUH	120/ 4,138	-0.5035	0.1894	13.7%	0.60 [0.42, 0.88]
**Total (95% CI)**					**0.53 [0.46, 0.61]**
Heterogeneity: Tau^2=0.00; Chi^2=0.85, df=2 (p=0.65); I^2=0.0%					
Test for overall effect: Z=9.06 (p<0.00001)					
**40% eGFR reduction**					
CGMH	406/ 20,992	-0.7782	0.1102	81.2%	0.46 [0.37, 0.57]
NCKUH	13/ 2,202	-0.8519	0.6063	2.5%	0.43 [0.13, 1.40]
NTUH	76/ 4,138	-0.7432	0.2472	16.3%	0.48 [0.29, 0.77]
**Total (95% CI)**					**0.46 [0.38, 0.56]**
Heterogeneity: Tau^2=0.00; Chi^2=0.03, df=2 (p=0.98); I^2=0.0%					
Test for overall effect: Z=7.80 (p<0.00001)					
**50% eGFR reduction**					
CGMH	255/ 20,992	-0.8686	0.1382	83.0%	0.42 [0.32, 0.55]
NCKUH	4/ 2,202	-0.6890	1.2195	1.2%	0.50 [0.05, 5.48]
NTUH	47/ 4,138	-0.8571	0.3170	15.8%	0.42 [0.23, 0.79]
**Total (95% CI)**					**0.42 [0.33, 0.54]**
Heterogeneity: Tau^2=0.00; Chi^2=0.02, df=2 (p=0.99); I^2=0.0%					
Test for overall effect: Z=6.86 (p<0.00001)					

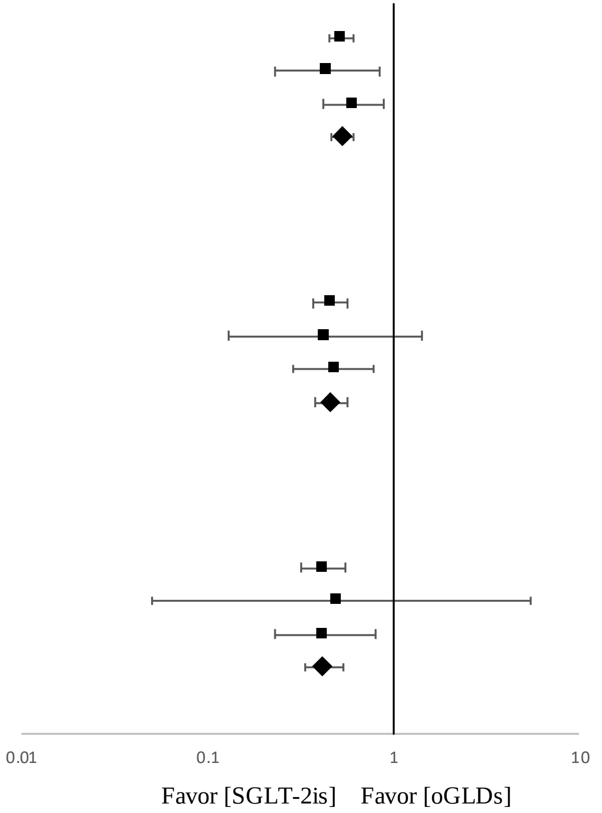

Abbreviation: CGMH: Chang Gung Memorial Hospital; CI: confidence interval; NCKUH: National Cheng Kung University Hospital; NTUH: National Taiwan University Hospital; SE: standard error.

Figure S6. Forest plots for 30%, 40%, and 50% estimated glomerular filtration rate (eGFR) reduction of sodium glucose cotransporter-2 inhibitor (SGLT-2i) versus other glucose-lowering drug (oGLD) use (on-treatment analysis) in overall study cohort from CGMH, NCKUH, and NTUH

Subgroup	Number of event/ total patients	log[Hazard ratio]	SE	Weight	Hazard ratio Random [95% CI]
**30% eGFR reduction**					
CGMH	220/ 20,992	-0.8354	0.1394	77.7%	0.43 [0.33, 0.57]
NCKUH	14/ 2,202	-1.2192	0.6065	4.1%	0.30 [0.09, 0.97]
NTUH	54/ 4,138	-0.6060	0.2884	18.2%	0.55 [0.31, 0.96]
**Total (95% CI)**					**0.45 [0.35, 0.57]**

Heterogeneity: Tau^2=0.00; Chi^2=0.99, df=2 (p=0.61); I^2=0.0%
Test for overall effect: Z=6.59 (p<0.00001)

Subgroup	Number of event/ total patients	log[Hazard ratio]	SE	Weight	Hazard ratio Random [95% CI]
**40% eGFR reduction**					
CGMH	99/ 20,992	-1.5450	0.2527	75.0%	0.21 [0.13, 0.35]
NCKUH	6/ 2,202	-1.8763	1.0784	4.2%	0.15 [0.02, 1.27]
NTUH	24/ 4,138	-0.9587	0.4788	20.8%	0.38 [0.15, 0.98]
**Total (95% CI)**					**0.24 [0.15, 0.37]**

Heterogeneity: Tau^2=0.00; Chi^2=1.35, df=2 (p=0.51); I^2=0.0%
Test for overall effect: Z=6.56 (p<0.00001)

Subgroup	Number of event/ total patients	log[Hazard ratio]	SE	Weight	Hazard ratio Random [95% CI]
**50% eGFR reduction**					
CGMH	57/ 20,992	-1.7839	0.3597	82.5%	0.17 [0.08, 0.34]
NCKUH	1/ 2,202				Not aviailable
NTUH	12/ 4,138	-1.6094	0.7725	17.5%	0.20 [0.04, 0.91]
**Total (95% CI)**					**0.17 [0.09, 0.33]**

Heterogeneity: Tau^2=0.00; Chi^2=0.04, df=1 (p=0.84); I^2=0.0%
Test for overall effect: Z=5.38 (p<0.00001)

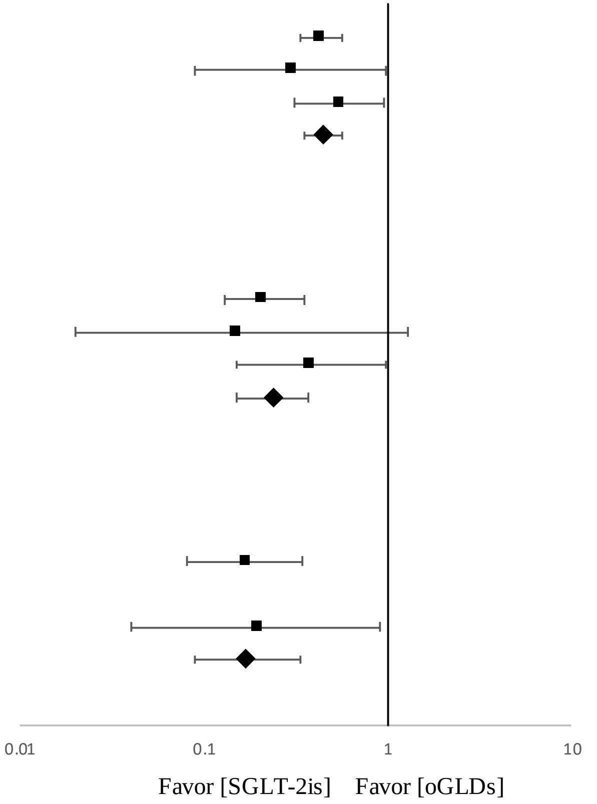

Abbreviation: CGMH: Chang Gung Memorial Hospital; CI: confidence interval; NCKUH: National Cheng Kung University Hospital; NTUH: National Taiwan University Hospital; SE: standard error.

Note: Because no patients reached 50% eGFR reduction in NCKUH, the hazard ratio estimate for this hospital site was not available.
